# Effect of Hyaluronic Acid on the Activity of Methylene Blue in Photogeneration of ^1^O_2_

**DOI:** 10.3390/molecules29225336

**Published:** 2024-11-13

**Authors:** Valeriya V. Kardumyan, Anastasia S. Kuryanova, Aleksandr V. Chernyak, Nadezhda A. Aksenova, Mikhail V. Biryukov, Nicolay N. Glagolev, Anna B. Solovieva

**Affiliations:** 1 N.N. Semenov Federal Research Center for Chemical Physics, Russian Academy of Sciences, Kosygin St. 4, 119991 Moscow, Russia; kuryanovaanastasi@gmail.com (A.S.K.); naksenova@mail.ru (N.A.A.); nikgl@mail.ru (N.N.G.); ann.solovieva@gmail.com (A.B.S.); 2 Federal Research Center of Problem of Chemical Physics and Medicinal Chemistry, Russian Academy of Sciences, Ac. Semenov Ave. 1, 142432 Chernogolovka, Russia; sasha_cherniak@mail.ru; 3 Scientific Center in Chernogolovka of the Institute of Solid State Physics Named Yu. A. Osipyan, Russian Academy of Sciences, Ac. Semenov Ave. 1, 142432 Chernogolovka, Russia; 4 Translational Medicine Research Center, Sirius University of Science and Technology, Olympic Ave. 1, 354340 Sochi, Russia; biryukov.mv@talantiuspeh.ru; 5 Faculty of Biology, Lomonosov Moscow State University, Leninskie Gori St. 1/12, 119234 Moscow, Russia

**Keywords:** methylene blue, hyaluronic acid, photogeneration of singlet oxygen, photooxidation of tryptophan, antibacterial photodynamic therapy

## Abstract

The effect of a natural polysaccharide (hyaluronic acid (HA)) on the photocatalytic activity of methylene blue (MB) was studied both under model conditions (a tryptophan photooxidation reaction in water) and with in vitro experiments on *P. aeruginosa* and *S. aureus* bacterial cultures. It was shown spectrophotometrically that, in the presence of HA, an increase in the optical density of the absorption bands λ = 665 nm and 620 nm—which correspond to the monomeric and dimeric forms of the dye, respectively—was observed in the EAS of the dye, while the ratio of the optical density of these bands remained practically unchanged. When adding HA to MB, the intensity of singlet oxygen ^1^O_2_ photoluminescence and the degree of fluorescence polarization of MB increase. The observed effects are associated with the disaggregation of molecular associates of the dye in the presence of HA. The maximum increase in the photocatalytic activity of MB (by 1.6 times) was observed in the presence of HA, with concentrations in a range between 0.0015 wt.% and 0.005 wt.%.

## 1. Introduction

The problem of resistance of microorganisms to antimicrobial drugs, including antibiotics, has become global. The WHO has included bacterial resistance in the list of the 10 most serious threats to humanity [1,2]. New strains of bacteria resistant to all or almost all known antibiotics are emerging. According to a meta-analysis published in the Lancet in 2019, bacterial resistance to antibiotics has become the third leading cause of death worldwide after ischemic heart disease and stroke [3]. Antibacterial photodynamic therapy (aPDT) could become a full-fledged alternative to antibiotic therapy in the treatment of complicated local infections of the skin and soft tissues, which are among the most dangerous pathologies and are often accompanied by systemic intoxication, resulting in severe sepsis and multiple organ failure that can lead to death in patients [4,5]. The method is based on the ability of photosensitizers (previously introduced into the affected tissues) to transfer excitation energy to molecular oxygen under irradiation conditions, which leads to the formation of reactive oxygen species (ROS) [6,7]. ROS destroy pathogenic microorganisms without causing the body to become accustomed to the treatment method [6,7,8]. Cationic photosensitizers (PS) (porphyrins, chlorins, and dyes), which are believed to more easily penetrate the cell wall of gram-positive and even the most bactericidal-resistant gram-negative bacteria compared to uncharged PS, are primarily used in aPDT [9,10]. A positive charge on the photosensitizer molecule increases the efficiency of the interaction between the photosensitizer and the bacterial cell (the target) [11]. Methylene blue (MB) is one of the actively studied cationic photosensitizers for aPDT [12]. This dye (quantum yield of singlet oxygen generation Φ_∆_~0.52) absorbs in the region of 660 nm [13]. Due to its ease of production, low preparation cost, bactericidal properties, and photostability, MB is approved and recommended as a PS in the treatment of periodontal disease, nail plates, and candidiasis by the aPDT method [14,15,16].

It should be noted that, to completely cure a local infectious diseases by the aPDT method, it is necessary not only to effectively inactivate pathogenic microorganisms but also to initiate regenerative processes in the wound. To solve this problem, photosensitizers are often used together with biologically active compounds (enzymes and growth factors), which can initiate the growth of new tissue in the wound [17,18,19]. One of the biologically active compounds that plays an important role in the wound healing process is hyaluronic acid (HA), the main component of the extracellular matrix of the skin, which affects the formation of fibrin clots, as well as the production and release of interleukins and cytokines, and stimulates the proliferation of fibroblasts and keratinocytes [20,21]. 

The main disadvantage of photosensitizers of various natures, which reduce the activity of PS in generating ROS, is their tendency to aggregate in aqueous solutions by forming dimers and larger associates with reduced photoactivity compared to monomers [22,23,24]. 

In this paper, the effect of HA on the photodynamic activity of methylene blue under model conditions and when exposed to bacterial cells was investigated.

## 2. Results and Discussion

**Figure 1:**
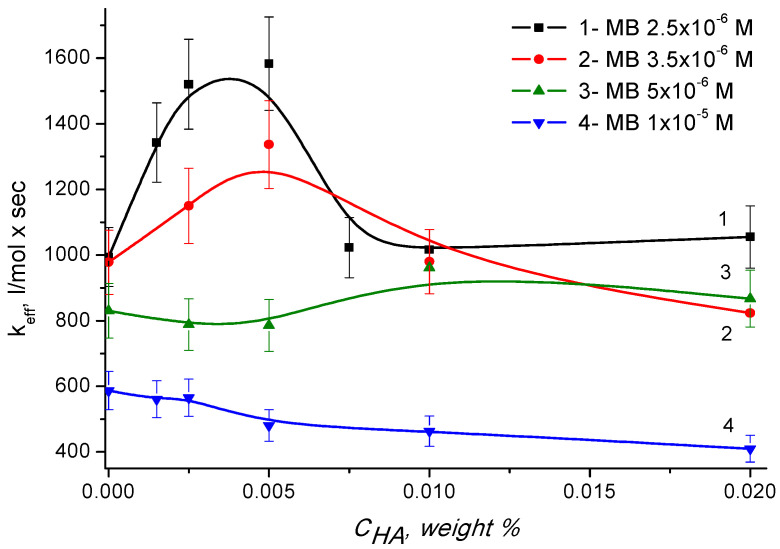
Dependence of the effective rate constant of tryptophan photooxidation (***k_eff_***), catalyzed by the methylene blue–hyaluronic acid (MB-HA) double systems on the concentration of HA. The concentration of MB is 2.5 × 10^−6^ M (curve 1); 3.5 × 10^−6^ M (curve 2); 5 × 10^−6^ M (curve 3); 1 × 10^−5^ M (curve 4); the concentration of tryptophan is 1.5 × 10^−5^ M.

**Scheme 1:**
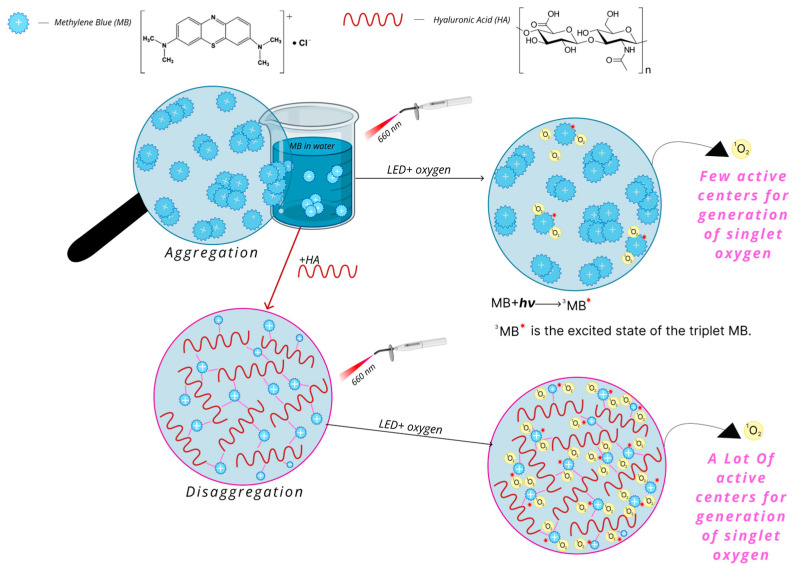
Schematic illustration of the processes occurring in the initial solution of MB and in the presence of HA under the action of light.

The dependence of the effective rate constant ***k_eff_*** of the tryptophan photooxidation reaction in the presence of MB on the concentration of HA is shown in Figure 1. The value of ***k_eff_*** and the trend ***k_eff_*** = ***f*(*C_HA_*)** both depend on the concentration of MB. Thus, at low concentrations of MB, the addition of HA leads to an increase in ***k_eff_*** of 1.1–1.6 times (Figure 1, curves 1–3). With an increase in the concentration of MB, the rate of the model reaction in the presence of HA decreases. Previously, we showed that, in the presence of another anionic polysaccharide, sodium alginate (SA), the photocatalytic activity of MB decreased regardless of the dye concentration due to the electrostatic interaction between the cationic MB and the carboxyl groups of SA [22]. It is known that HA, having a negative charge due to the presence of carboxyl groups, can also form complex compounds with some cationic dyes, such as Azure A [25] and Alcian blue [26]. Since the primary structure of HA consists of repeating disaccharide units of D-glucuronic acid and *N*-acetyl-d-glucosamine, HA can also form intra- and intermolecular hydrogen bonds (up to five hydrogen bonds in each unit of the macromolecule) [27]. Most likely, HA can form such donor–acceptor complexes with MB molecules, promoting their disaggregation [28,29,30]. Jiao et al. [28,29,30] noted that the interaction of positively charged dyes with negatively charged glycosaminoglycans can lead to a change in the conformation of the dye, which in turn leads to hydrophobic interactions between the dye molecules bound to the glycosaminoglycans. Thus, the increase in the photocatalytic activity of MB (C = 2.5 × 10^−6^–5 × 10^−6^ M) in the presence of low concentrations of HA (≤0.1 wt. %) is possibly associated with the disaggregation of MB. A schematic illustration of this process is shown in Scheme 1. Previously, a similar effect, leading to the disaggregation of photosensitizers (photoditazine, methylene blue, and rose bengal) and an increase in the ***k_eff_*** value, was discovered when using amphiphilic polymers (PVP and pluronic) in a reaction of photocatalytic oxidation of tryptophan in water [22,31,32]. The curves exhibit extrema, possibly due to the fact that, with a further increase in the concentration of the polymer in water, the degree of ineffective adsorption of the dye and substrate increases, and the polymer, in this case, acts as a “diluent” that prevents contact between MB and tryptophan, since tryptophan practically does not interact with HA. Due to the short lifetime of ^1^O_2_ in aqueous solutions, this leads to a decrease in the value of ***k_eff_***.

These conclusions are confirmed by an increase in the intensity of photoluminescence of singlet oxygen ^1^O_2_ in the presence of MB upon the introduction of HA into the system (Table 1).

### 2.1. Electronic Absorption Spectra of MB and MB-HA

The interaction between methylene blue and hyaluronic acid is also reflected in the electronic absorption spectra (EAS) of the dye [33].

**Figure 2:**
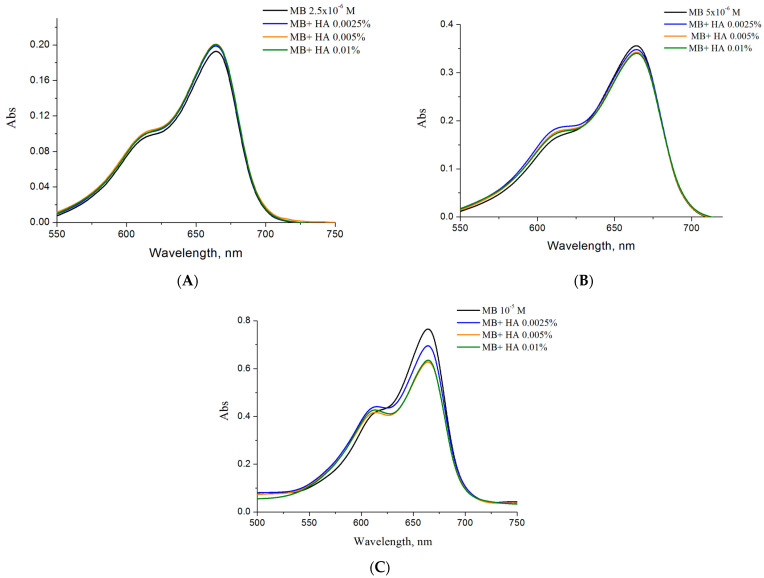
Electronic absorption spectra of MB: [MB] = 2.5 × 10^−6^ M ((**A**) curve, black), [MB] = 5 × 10^−6^ M ((**B**) curve, black), [MB] = 10^−5^ M ((**C**) curve, black) and MB-HA at different concentrations of HA: [HA] = 0.0025 wt. % ((**A**–**C**) curves, blue), [HA] = 0.005 wt. % ((**A**–**C**) curves, yellow), [HA] = 0.01 wt. % ((**A**–**C**) curves, green).

Figure 2 shows the EAS of MB in water at different concentrations of HA and dye (Figure 2A–C). The absorption spectrum of MB contains an intense (ε~6.5 × 10^4^ M^−1^ × cm^−1^) asymmetric absorption band with λ = 665 nm (**D_max_**), corresponding to the monomeric form of the dye, and a small shoulder in the region of λ = 610–615 nm (**D_shoulder_**), which is attributed to the dimeric form of the dye [34]. It is likely that, when adding HA to an aqueous solution of MB with a concentration of 2.5 × 10^−6^ M (Figure 2A), an increase in the optical density of both absorption bands (λ = 665 nm and 620 nm) in the EAS of the dye is observed. This is associated with the interaction of HA with both the monomeric and dimeric forms of MB. Moreover, the almost unchanged value of *φ =* **D_max_/D_shoulder_** confirms this (Table 2).

From Figure 2, it follows that, at a concentration of MB greater than or equal to 5 × 10^−6^ M, the addition of HA leads to a drop in the optical density of the main band λ = 665 and a slight increase in the shoulder, with λ = 620 nm in the EAS (Figure 2B,C), which indicates some aggregation of the dye molecules. This is also reflected in a drop in the φ value (Table 2).

It should be noted that the fluorescence spectra of MB in the presence of HA remain almost unchanged, also indicating an insignificant effect of HA on the aggregation processes of MB in aqueous solutions (in contrast to polyanionic SA [22]).

### 2.2. Fluorescence Anisotropy

To identify the interaction between MB and HA molecules, the effect of the polymer on the anisotropy (polarization) of methylene blue fluorescence was studied (Table 3). The degree of fluorescence polarization at [MB] = 2.5 × 10^−6^ M in the initial aqueous solution is ***r*** = **0.087**, which is consistent with literature data [35].

In the presence of HA, the ***r*** value for the dye increases (up to **0.095**). It is probable that the increase in the degree of luminescence polarization is due to the interaction of the fluorophore functional groups with fragments of the polymer structure, leading to a limitation of the fluorophore freedom of rotation in an aqueous solution and an increase in the ***r*** value. This effect is most pronounced at an HA content of 0.005%, similar to what was observed when studying kinetic data (Figure 1).

### 2.3. ^1^H-NMR

**Figure 3:**
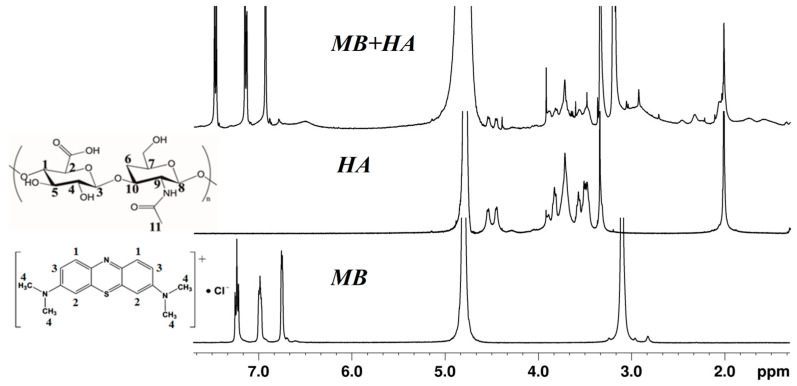
^1^H-NMR spectra of MB, HA, and MB-HA (1:1 by mass) in D_2_O at 22.4 °C.

To confirm the possible interaction of methylene blue with hyaluronic acid, ^1^H-NMR spectra of the original dye, the polysaccharide, and their mixture in a 1:1 ratio by weight were studied. As can be seen from Table 4 and Figure 3, for the MB-HA system, a change in the position of the proton signals is observed for both the dye molecules and for the polysaccharide molecules. In the presence of HA, all signals of the aromatic region of the dye are shifted to weak fields by 0.2 ppm. At the same time, it seems that the HA signals in the spectrum did not change much in their position or shape with the addition of MB. However, several very wide signals can be seen in the region of 1.5–3.5 ppm (HA signals shifted to strong fields). Most likely, some of the HA molecules form stable complexes with MB. The shielding of HA molecules occurs due to the aromatic part of the MB molecules, and the acid signals are shifted to strong fields.

### 2.4. FT-IR

**Figure 4:**
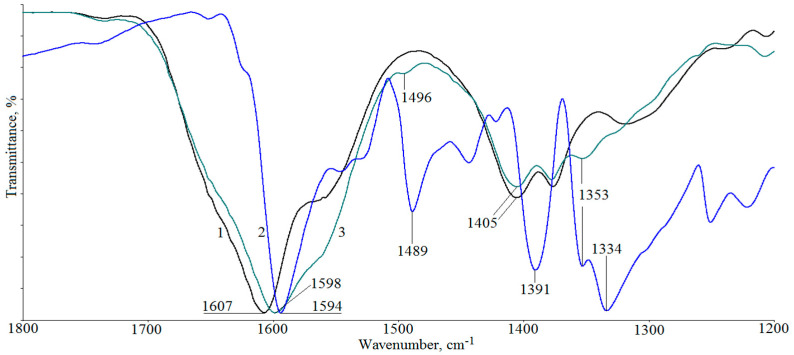
FT−IR spectra of HA (1), MB (2), and MB-HA (1:4 by mass) (3).

Figure 4 shows fragments of the IR spectra of HA in the absence and presence of the dye. In the IR spectra of HA (curve 1, Figure 4), the intense bands are observed at 1607 cm^−1^ and 1405 cm^−1^, corresponding to vibrations of the COO- groups and the C-O-, OH, and CH groups [36]. In the IR spectra of MB (curve 2, Figure 4), the intense bands are observed at 1594 cm^−1^, 1489 cm^−1^, 1391 cm^−1^, 1353 cm^−1^, and 1334 cm^−1^, which can be attributed to the vibrations of the C=N, C=C, C=S+, CH, and C-N groups, respectively [37]. In the MB-HA system (1:4 ratio by weight) (curve 3) a shift of the band from 1607 cm^−1^ to 1598 cm^−1^, corresponding to vibrations of the COO- groups in HA macromolecules, was observed. Since there is an intense band at 1594 cm^−1^ in the same region, corresponding to the vibrations of the C=N and C=C groups in the dye structure, it can be assumed that the band at 1598 cm^−1^ in the spectra of the MB-HA systems is a superposition of the bands at 1607 cm^−1^ and 1594 cm^−1^. However, the shift of the band at 1489 cm^−1^ (to 1496 cm^−1^) possibly indicates an interaction of the functional groups of the dye with HA macromolecules. In this regard, it can be assumed that the shift of the COO- band from 1607 cm^−1^ to 1598 cm^−1^ is also due to the ionic interaction of the carboxylate ions of the HA macromolecules with the MB molecules.

### 2.5. Photodynamic Activity of MB and MB-HA

Preliminary studies were conducted to determine the photodynamic activity of the dye against pathogens in the absence and presence of HA. To evaluate the effectiveness of the dye and the MB-HA system in inactivating bacteria, the traditional method of counting bacteria in a Petri dish was used. *S. aureus* (gram-positive) and *P. aeruginosa* (gram-negative) were used as bacteria.

**Figure 5:**
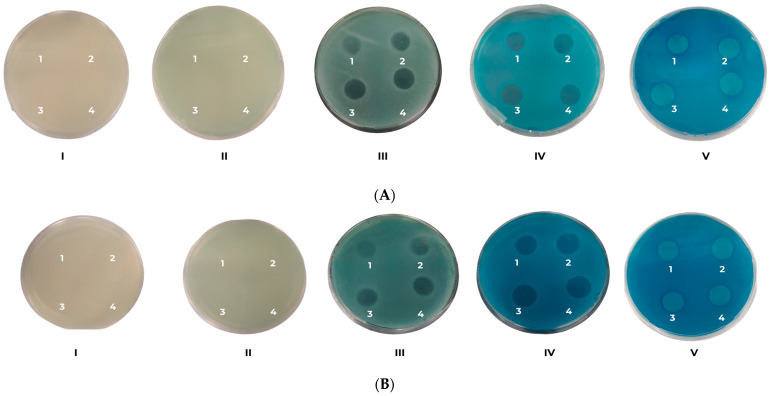
Photodynamic inactivation of *S. aureus* (**A**) and *P. aeruginosa* (**B**) using methylene blue in different concentrations and in combination with visible light at 660 nm, where C(MB): I—10^−6^, II—5 × 10 ^−6^, III—10^−5^, IV—5 × 10^−5^, and V—10^−4^ M. Light dose: 1—50 J/cm^2^, 2—70 J/cm^2^, 3—85 J/cm^2^, and 4—100 J/cm^2^.

**Figure 6:**
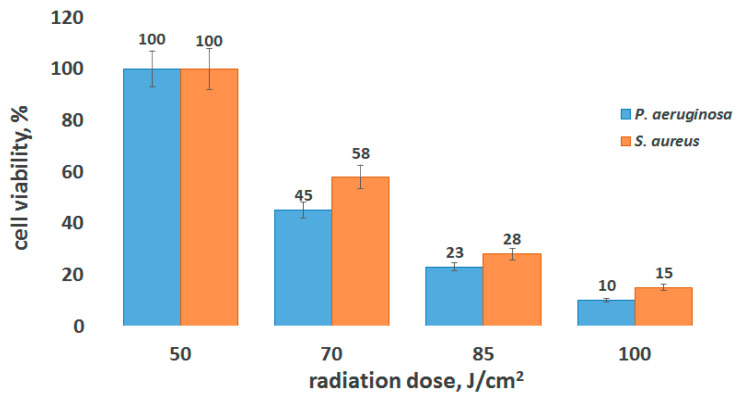
Survival diagram of *S. aureus* and *P. aeruginosa* bacteria treated with 10^−5^ M of MB in combination with visible light at 660 nm and with various radiation doses.

These bacteria are the main causative agents of superficial infections and belong to the ESKAPE group (*Enterococcus faecium, Staphylococcus aureus, Klebsiella pneumoniae, Acinetobacter baumannii, Pseudomonas aeruginosa*, and *Enterobacter*) of deadly pathogens resistant to antibiotics [38,39]. Bacterial colony-forming units are shown in Figure 5 and Figure 6.

First, the photodynamic activity of methylene blue against *S. aureus* and *P. aeruginosa* was studied at different dye concentrations and light doses (Figure 5A,B). As can be seen from Figure 5A,B, none of the radiation doses used suppressed bacterial growth on the plates with MB concentrations of 10^−6^ M and 5 × 10^−6^ M. The suppression of bacterial growth was observed when irradiating dishes with MB concentrations above 10^−5^ M. Moreover, bacterial survival decreased with an increasing radiation dose (Figure 6). As can be seen from Figure 6, in order to inactivate more than 85–90% of microbes, it is necessary to use a radiation dose of 100 J/cm^2^. In this case, *P. aeruginosa* is most sensitive to aPDT at a given dye concentration of 10^−5^ M and radiation doses of 70–100 J/cm^2^. In previous studies [40,41,42], it was shown that cationic PS, capable of effectively binding to the outer membrane and displacing Ca^2+^ and Mg^2+^ ions (which are necessary for the vital activity of pathogens), are preferable for the inactivation of *P. aeruginosa.* Zada et al. demonstrated the effectiveness of methylene blue at different concentrations against *P. aeruginosa* [43]. Moreover, a maximum reduction in bacteria of 3.48 log_10_ and 4.32 log_10_ was observed when using MB together with a laser diode with a wavelength of 635 nm at a radiation dose of 90 J/cm^2^ and 108 J/cm^2^.

As can be seen from Figure 5, a further increase in the concentration of PS up to 5 × 10^−5^ M and 10^−4^ M leads to a complete suppression in growth of both bacteria in the irradiation zones at all radiation doses used.

Thus, MB exhibits antimicrobial phototoxic activity against *S. aureus* and *P. aeruginosa* at a concentration of 10^−5^ M and a radiation dose of 70 J/cm^2^. These concentrations of the cationic dye and radiation dose were chosen as working parameters for further studies.

**Figure 7:**
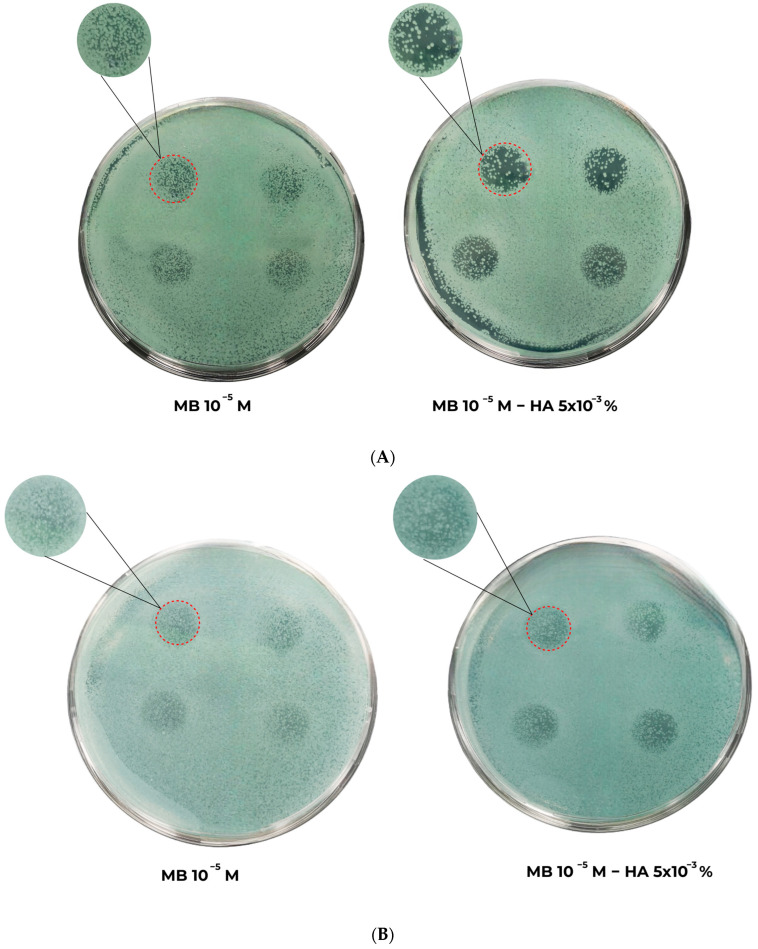

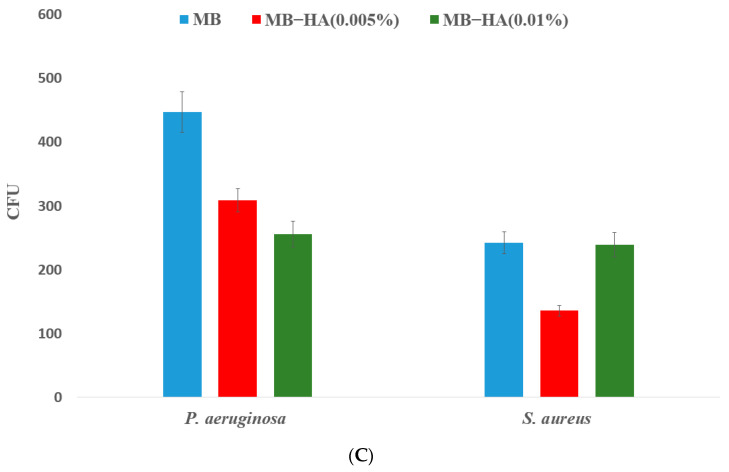
Zones of growth inhibition caused by the irradiation of *S. aureus* (**A**) and *P. aeruginosa* (**B**) with a 660 nm LED on an LB medium with added MB or MB-HA. C_MB_ = 10^−5^ M. Radiation doses: 70 J/cm^2^. Irradiation was performed in quadruple repetition. The circles indicate the inhibition zone, magnified 1.5 times. (**C**) Diagram of the colony-forming units (CFUs) after the antimicrobial photodynamic therapy (aPDT) treatment of *P. aeruginosa* and *S. aureus* with MB and MB-HA with visible light at 660 nm and a radiation dose of 70 J/cm^2^. C_MB_ = 10^−5^ M and C_HA_ = 0.005 wt. % or 0.01 wt. %. Data are presented as the mean ± standard deviation (*n* = 4).

The MB-HA system also exhibited photodynamic activity against both bacteria. Figure 7A,B shows digital images of *S. aureus* and *P. aeruginosa* culture plates after aPDT treatment using MB and MB-HA, where the HA concentration was set to 0.005%. The use of the MB-HA system in combination with light irradiation led to a 1.78-fold reduction in *S. aureus* colonies and a 1.45-fold reduction in *P. aeruginosa* colonies compared to the use of the original dye alone against these bacteria (Figure 7C). In this case, *S. aureus* is more sensitive to photoinactivation in the presence of the MB-HA system. As mentioned above, this difference is associated with both the bacteria’s structure and, apparently, with the formation of a complex between MB and HA.

It is interesting to note that when the concentration of HA is increased to 0.01%, the indicated effect of HA on the activity of MB against both bacterial strains is not detected. As noted earlier (Figure 1), a similar concentration dependence of the effect of HA on the activity of MB in the photogeneration of singlet oxygen (in the reaction of tryptophan photooxidation) was observed under model conditions.

## 3. Materials and Methods

### 3.1. Reagents

Methylene blue (MB), a cationic dye of a phenothiazine nature (3,7-bisdimethylaminophenothiazine chloride, MM 373.90 g/mol, extinction coefficient at a wavelength of 665 nm (ε_665nm_) = 64,800 M^−1^ × cm^−1^) (Figure 3), was used as a photosensitizer (Chimmed, Moscow, Russia). Hyaluronic acid (HA) was used in the form of sodium salt (MM 39,000 Da, Evergrowing, Nanjing, China) (Figure 3). d,l-tryptophan (Trp) (MM 204.23 g/mol, Acros organic, Geel, Belgium) was used as a substrate in the photooxidation reaction.

### 3.2. Preparation of the MB-HA System and Study of Its Photosensitizing Properties in the Generation of Singlet Oxygen

To prepare the reaction system, methylene blue and hyaluronic acid were dissolved in water. The concentration of MB varied from 2.5 × 10^−6^ to 1 × 10^−5^ M, and the concentration of HA was in the range of 0–0.02 wt. %.

The photosensitizing activity of MB in the presence of a polysaccharide during the generation of singlet oxygen was determined in a model reaction of tryptophan photooxidation in an aqueous solution. Trp was added to the resulting reaction systems. The concentration of Trp was 1.5 × 10^−5^ M. The solutions of components were mixed in certain ratios for 15 min, and the order of mixing did not affect the activity of the system. The reaction of Trp photooxidation with atmospheric oxygen was carried out in a quartz cuvette (V = 3 mL, thickness = 10 mm). Irradiation was performed using an AFS LED phototherapeutic device (Polironik, Moscow, Russia) with a wavelength of 660 nm and a power of 1100 mW, while stirring with a magnetic stirrer. The kinetics of the substrate photooxidation process were recorded by a decrease in the intensity of the band at λ_em_ = 356 nm in the tryptophan fluorescence spectrum (excitation wavelength λ_ex_ = 280 nm).

For the comparative assessment of the photosensitizing activity of the dyes and their complexes with AP and PS, the effective constant *k_eff_* of the photooxidation rate of tryptophan was used, which was calculated using the equation that follows:

$$k_{eff}=\frac{I_{0}-I_{t}}{I_{0}\times \Delta t}\times \frac{1}{C_{MB}}$$

where *I*_0_ and *I_t_* are the initial tryptophan fluorescence intensity and the substrate fluorescence intensity at time *t* and *C_MB_* is the concentration of methylene blue. The number of measurements was no less than five, and the measurement error was no more than 10%.

### 3.3. Spectral Research 

The EAS of methylene blue in the studied solutions in the absence and presence of HA was recorded using a Cary 50 spectrophotometer (Varian, Belrose, Australia); the fluorescence spectra of MB and the MB-HA system were studied using a Cary Eclipse spectrofluorometer (Varian, Australia) (excitation wavelength λ_ex_(MB) = 665 nm). Singlet oxygen luminescence spectra were recorded using a Horiba Fluoromax Plus spectrofluorometer (Horiba-Jobin-Yvon, Palaiseau, France), a DSS-IGA020L IR detector (spectral range 800–1700 nm), and a TLP RG780 long-wave filter (Edmund Optics, Barrington, NJ, USA). 

The degree of fluorescence anisotropy (polarization) (*r*) of the original MB, as well as the MB-HA system, was automatically calculated by a Cary Eclipse spectrofluorometer (Varian, Australia) based on the values of G, I_VV_, and I_VH_ in the fluorescence emission spectra of the studied solutions using the equation presented in the publication by Kuryanova et al. [44].

To determine *r*, the studied solutions were excited by light with a wavelength of λ_ex_(MB) = 665 nm. The anisotropy of the reaction mixtures was recorded at a wavelength of λ_MB_ = 685 nm. The measurement error was no more than 3–5%, and the number of measurements in one sample was 5 to 8.

NMR measurements were performed using the equipment of the Multi-User Analytical Center of the Federal Research Center of Problems of Chemical Physics and Medicinal Chemistry. The ^1^H-NMR was recorded on a Bruker AVANCE III 500 MHz high-resolution spectrometer (Ettlingen, Germany) at room temperature (22.4 °C) with an operating frequency of 500 MHz. The dye (MB), polysaccharide (HA), and their mixture (weight ratio of 1:1) were each dissolved in D_2_O (Sigma-Aldrich, Burlington, MA, USA, 99 atom % D) and placed in standard glass ampoules (outer diameter 5 mm). The chemical shift scale was calibrated using the residual protons of the solvent signal (H_2_O, δH = 4.80 ppm).

The FT-IR analysis of the samples (HA, MB, and MB-HA (1:4 by mass)) was performed using a Spectrum Two FT-IR Spectrometer (PerkinElmer, Waltham, MA, USA) in attenuated total reflectance (ATR) mode. The spectrometer features were as follows: high-performance, room temperature LiTaO_3_ MIR detector and a standard optical system with KBr windows for data acquisition over a spectral range of 4000–350 cm^−1^, with a resolution of 0.5 cm^−1^. Spectra were normalized by the band ~1600 cm^−1^.

### 3.4. Photodynamic Activity of MB and MB-HA 

The strains used in this work were *P. aeruginosa* (*Pseudomonas aeruginosa*) ATCC 27853 and *S. aureus* (*Staphylococcus aureus*) ATCC 29213. Maintenance of the bacterial cultures and experimental setup were carried out using an LB Miller medium (VWR Life Science, Radnor, PA, USA) in liquid form or with the addition of agar (bacteriology grade, AB EUR (DiaM, Moscow, Russia)), when the use of solid media was required.

For photodynamic inactivation using MB and MB-HA, two pathogen strains were selected: gram-negative bacteria *P. aeruginosa* and gram-positive bacteria *S. aureus*. 

The PS system (MB or MB-HA) was added to the melted nutrient medium, which was poured into Petri dishes. Afterward, a bacterial lawn was applied to the surface of the solidified media using a sterile cotton swab. Then the Petri dishes were exposed at room temperature in a dark laminar flow hood for 20–30 min. The resulting samples were subjected to photoirradiation using an LED phototherapeutic device with a wavelength of λ = 660 nm and a power of 1100 mW. The radiation dose was 50–100 J/cm^2^, and the exposure time was 2–4.5 min. The concentration of MB was between 10^−6^ and 10^−4^ M, and the concentrations of HA were 0.005 wt. % and 0.01 wt. %.

The samples were incubated at 37 °C for 24 h. Analysis was carried out by counting colony-forming units (CFUs) in irradiation zones (light spots), or if colonies were not observed, a complete growth inhibition was stated.

## 4. Conclusions

Thus, in this work, it was demonstrated that the activity of methylene blue in the photogeneration of singlet oxygen (in the reaction of tryptophan photooxidation) increases in the presence of hyaluronic acid. This was confirmed by the increase in the intensity of photoluminescence of singlet oxygen ^1^O_2_ in the presence of MB upon the addition of HA. This may be due to the disaggregation of molecular aggregates in the dye in the presence of HA since the optical density of the absorption band at 665 nm in the EAS increased in the presence of HA. The NMR method showed that, in a joint solution of MB and HA, the signals of protons in the aromatic region of the dye shift to weak fields, and the signals of protons in hyaluronic acid shift to strong fields, which indicates the formation of sufficiently strong MB-HA complexes. The effect of HA on the photocatalytic activity of the dye was most pronounced at a concentration of MB of 2.5–3.5 × 10^−6^ M. A maximum increase in the photocatalytic activity of MB (by 1.3–1.6 times) was observed in the presence of HA at concentrations in a range between 0.0015 wt.% and 0.005 wt.%. The MB-HA complex can be used in antimicrobial photodynamic therapies due to its high photocatalytic activity and accessibility. An increase in the MB’s bactericidal activity against *S. aureus* and *P. aeruginosa* bacteria in the presence of HA was shown.

## Figures and Tables

**Table 1:** The luminescence peak area of ^1^O_2_ singlet oxygen when using MB and the MB-HA as photosensitizers in D_2_O at 293 K at different HA concentrations.

[HA], wt. %	S_lum 1276 nm,a.u._
0	0.1879 ± 0.00245
0.0015	0.22185 ± 0.00165
0.0025	0.22185 ± 0.00715

**Table 2:** The ratio *φ =* **D_max_/D_shoulder_** at different MB concentrations.

	C_MB_	2.5 × 10^−6^ M	5 × 10^−6^ M	1 × 10^−5^ M
C_HA_				
0		2.0	2.1	1.9
0.0025%		1.9	1.9	1.6
0.0050%		1.9	1.9	1.5
0.0100%		1.9	1.9	1.5

**Table 3:** Fluorescence anisotropy (*r*) at [MB] = 2.5 × 10^−6^ M in the absence and presence of HA. The excitation wavelength is 665 nm.

Sample	*r* of the Samples in Aqueous Solution (685 nm)
MB	0.087 ± 0.003
MB+HA (0.005%)	0.095 ± 0.004
MB+HA (0.010%)	0.090 ± 0.007

**Table 4:** Proton chemical shifts for HA, MB, and MB-HA (1:1 by mass) in D_2_O at 22.4 °C.

Position of Protons in the MB Molecule	MB Proton Signals, ppm	Position of Protons in the HA Molecule	HA Proton Signals, ppm	MB Proton Signals in the Presence of HA, ppm	HA Proton Signals in the Presence of MB, ppm
1	7.14 7.24	3, 8	3.84 4.55 3.68 4.46	7.40 7.47	-
3	6.89 6.99	2–7, 9, 10	3.82 3.93 3.73 3.84 3.63 3.73 3.47 3.57 3.42 3.52 3.37 3.48 3.24 3.35	7.06 7.15	3.82 3.92 3.62 3.72 3.38 3.49 3.24 3.35
2	6.66 6.75			6.83 6.94	
4	3.01 3.10	11	1.91 2.02	3.10 3.20	1.91 2.02

## Data Availability

Data are contained within the article.
